# Impact of postoperative weight loss on anticoagulant dosing after major lower limb amputation

**DOI:** 10.1308/rcsann.2025.0120

**Published:** 2026-01-20

**Authors:** A Al-Kassar, A Okram, K Poluru, NB Teo

**Affiliations:** Countess of Chester Hospital, UK

**Keywords:** Postoperative weight loss, Major lower limb amputation, Anticoagulant dosing

## Abstract

**Objective:**

Major lower limb amputation is often the final treatment for patients with chronic limb-threatening ischaemia. Postoperative weight loss is a common but under-recognised issue that may affect dosing accuracy for weight-dependent anticoagulants such as low molecular weight heparin (LMWH) and direct oral anticoagulants (DOACs). This study assessed the extent of postoperative weight change and the frequency of anticoagulant dose adjustment in routine practice.

**Methods:**

A single-centre retrospective cohort study included patients who underwent above- or below-knee amputation between July 2022 and January 2023. Inclusion criteria were documented pre- and postoperative weights and inpatient prescription of LMWH or DOACs. LMWH was prescribed exclusively as dalteparin and DOAC therapy as edoxaban. Doses were reviewed against British National Formulary weight-based recommendations.

**Results:**

Of 39 patients, 17 met inclusion criteria (median age 69 years; 53% male). Procedures were divided evenly between emergency and elective. The mean postoperative weight loss was 7.5%±2.3% of preoperative body weight. Patients undergoing above-knee amputation lost more weight (mean 8.7kg) than those with below-knee amputation (mean 3.8kg). Two patients (11.8%) required dose adjustments – one on edoxaban after falling below 60kg, and one on dalteparin after dropping from 59.5kg to 55.5kg.

**Conclusions:**

Major amputation is associated with clinically relevant postoperative weight loss, yet anticoagulant dose adjustments were uncommon. Routine postoperative weight monitoring and prompt review of weight-based therapy are recommended to improve dosing accuracy and reduce potential anticoagulation risk.

## Introduction

Major lower limb amputation is often the last resort for patients with chronic limb-threatening ischaemia (CLTI), particularly when revascularisation is no longer possible or has failed. These procedures – either above-knee amputations (AKAs) or below-knee amputations (BKAs) – are typically performed in a high-risk population with significant comorbidities. While amputation can be necessary to alleviate pain or control infection, it also brings about substantial physiological and functional changes.^1^

Weight loss is a well-recognised consequence following major lower limb amputation, often resulting from a combination of muscle mass reduction, prolonged immobility, extended hospitalisation and nutritional changes during recovery. These factors, along with postoperative catabolic stress, can significantly alter a patient’s bodyweight. For individuals receiving weight-dependent medications – particularly anticoagulants such as low molecular weight heparin (LMWH) and direct oral anticoagulants (DOACs) – these changes can directly impact the accuracy and safety of dosing. Failure to adjust doses in response to postoperative weight loss may lead to underanticoagulation, increasing the risk of thromboembolic events, or overanticoagulation, heightening the potential for bleeding complications.^2–4^

Despite the potential impact on patient safety, weight monitoring and medication adjustment following major amputation are not always standard practice. Current clinical guidelines do not specifically address this issue, and there is limited evidence in the literature evaluating how frequently such changes are recognised and acted upon.

This study was conducted to assess postoperative weight changes in patients undergoing major lower limb amputations and to determine whether their anticoagulant doses were reviewed and adjusted appropriately during the postoperative period.

## Methods

A retrospective observational study was conducted at single vascular unit over a seven-month period, from July 2022 to January 2023. The study population included patients who underwent major lower limb amputation, defined as either AKA or BKA, during the study period.

Inclusion criteria were:

1. Documented preoperative and postoperative bodyweights, and
2. Prescription of either LMWH or DOACs during the inpatient stay.

Patients were excluded if they lacked complete weight documentation or were not prescribed LMWH or DOACs.

Data collected included demographic characteristics (age, sex), urgency of surgery (emergency versus elective), type of anticoagulant used and weight measurements recorded pre- and postoperatively. The percentage of weight change was calculated for each patient. Anticoagulant doses were reviewed in relation to postoperative weight and compared with the dosing recommendations outlined in the British National Formulary (BNF) guidelines.

LMWH was uniformly prescribed as dalteparin, administered at either prophylactic or therapeutic doses according to clinical indication. All patients receiving a DOAC were treated with edoxaban.

Institutional board approval was not required as the study was registered as an audit.

## Results

A total of 39 patients underwent major lower limb amputation during the study period. Of these, 17 met the inclusion criteria, having documented preoperative and postoperative weights and being prescribed either LMWH or DOACs.

Table 1 summarises the characteristics of the included patients. The median age was 69 years (±9), with a nearly even sex distribution (9 male and 8 female). The procedures were divided evenly between emergency (*n*=9) and elective (*n*=8) cases.

**Table 1:** Patient demographics, anticoagulant type and postoperative weight change

Pt ID	Sex	Age (years)	Amputation	Urgency	Anticoagulant	Dose type	Preoperative weight (kg)	Postoperative weight (kg)	Weight loss (kg)	Change (%)	Dose adjustment notes
1	M	68	AKA	Emergency	Dalteparin	Therapeutic	78	69	9	11.5	No change – still within 69–82kg band (15,000 IU)
2	F	71	AKA	Elective	Edoxaban	Fixed	63	59	4	6.3	Dose reduced (<60kg threshold)
3	M	67	AKA	Emergency	Dalteparin	Prophylactic	76	70	6	7.9	No change
4	F	74	AKA	Elective	Dalteparin	Therapeutic	80	72	8	10.0	No change
5	M	70	AKA	Emergency	Edoxaban	Fixed	85	77	8	9.4	No change
6	F	69	AKA	Elective	Dalteparin	Therapeutic	79	71	8	10.1	No change
7	M	63	AKA	Emergency	Dalteparin	Therapeutic	81	72	9	11.1	No change
8	F	75	AKA	Elective	Edoxaban	Fixed	77	71	6	7.8	No change
9	M	70	AKA	Emergency	Dalteparin	Therapeutic	83	74	9	10.8	No change – remains ≥83kg band
10	F	73	BKA	Elective	Dalteparin	Prophylactic	72	68	4	5.6	No change
11	M	66	BKA	Emergency	Edoxaban	Fixed	79	74	5	6.3	No change
12	M	68	BKA	Elective	Dalteparin	Prophylactic	60	56	4	6.7	Dose reduced (from 12,500 → 10,000 IU)
13	F	70	BKA	Emergency	Dalteparin	Therapeutic	74	70	4	5.4	No change
14	M	71	BKA	Elective	Edoxaban	Fixed	78	74	4	5.1	No change
15	F	65	BKA	Emergency	Dalteparin	Prophylactic	73	70	3	4.1	No change
16	M	72	BKA	Elective	Dalteparin	Therapeutic	80	77	3	3.8	No change
17	F	69	BKA	Emergency	Edoxaban	Fixed	77	74	3	3.9	No change

AKA = above-knee amputation; BKA = below-knee amputation; IU = international units; Pt = patient

Of the included patients, 5 were prescribed the DOAC edoxaban, and the remaining 12 received dalteparin (LMWH), either at prophylactic (*n*=5) or therapeutic (*n*=7) doses based on individual thrombotic risk and renal function.

On average, patients lost 13% (±8%) of their bodyweight following amputation. This corresponded to a mean postoperative loss of 8.7kg (range 5–13.8kg) for AKA and 3.8kg (range 2.5–5.3kg) for BKA. Despite this, only 2 of the 17 patients (11.8%) required anticoagulant dose adjustments in accordance with updated weight and BNF recommendations. One patient receiving edoxaban required a dose reduction after their postoperative weight fell below 60kg, and another receiving dalteparin required a reduction after their weight decreased from 60kg to 56kg.

**Figure 1:**
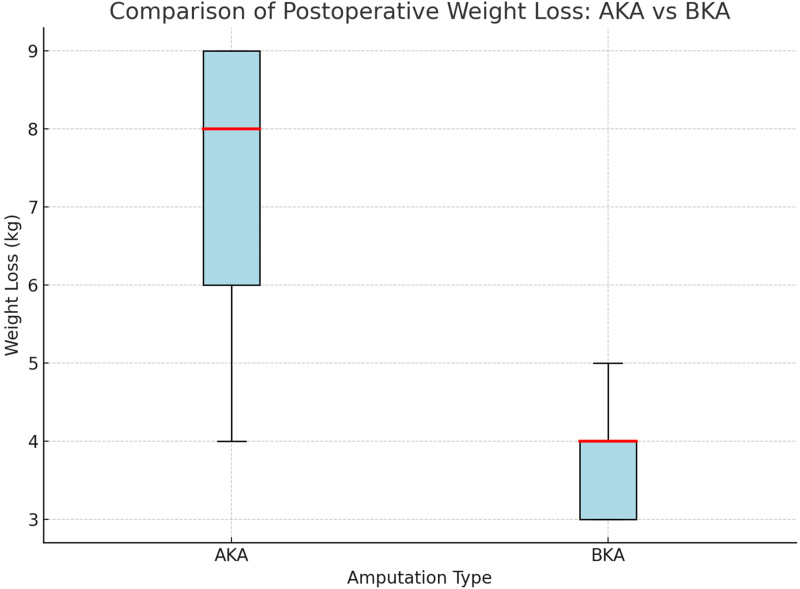
Comparison of postoperative weight loss between AKA and BKA. Patients undergoing AKA experienced greater postoperative weight loss than those with BKA, as shown by the higher median and broader interquartile range. The horizontal red line inside each box shows the median (50th percentile). AKA = above-knee amputation; BKA = below-knee amputation

As illustrated in Figure 1, patients who underwent AKA demonstrated significantly greater postoperative weight loss compared with those who had BKA.

This suggests that, while postoperative weight loss was common, dose adjustments were infrequent, potentially exposing patients to risks of under- or overanticoagulation. No clinical outcomes data (bleeding or thrombosis) were recorded, which limits assessment of direct clinical impact.

## Discussion

This study highlights a clinically significant but often under-recognised issue following major lower limb amputation: postoperative weight loss and its impact on dosing accuracy for weight-dependent anticoagulants. In our cohort, patients experienced a mean weight loss of 13% (±8%) after surgery, corresponding to an average loss of approximately 8.7kg after AKA and 3.8kg after BKA. Although only two patients (11.8%) required anticoagulant dose adjustments based on these postoperative weight changes, the findings suggest that even modest reductions in bodyweight can influence medication safety – particularly for dalteparin and edoxaban, where dosing accuracy is partially weight dependent.^2,3^

Anticoagulation therapy is essential in vascular patients, who are at increased risk of thromboembolic events due to immobility, comorbid conditions and surgical stress. Both underdosing and overdosing carry substantial risks – thrombosis in the former and bleeding in the latter.^5,6^

For LMWH, therapeutic dosing typically follows a milligrams per kilogram calculation, and inappropriate dosing can significantly affect drug efficacy and safety profiles. In this context, postoperative weight loss of 10–15% – as observed in our cohort – can be clinically meaningful.

Even modest reductions in weight may result in higher plasma concentrations of LMWH, while fixed-dose regimens such as edoxaban can lead to relative overdosing when body weight falls below 60kg. These findings are consistent with pharmacokinetic studies showing altered anticoagulant exposure in patients at extremes of weight.^2^

Routine weight reassessment and corresponding dose review should therefore form part of postoperative care, particularly in high-risk vascular patients undergoing major amputation.

In our cohort, dalteparin was the sole LMWH used, while edoxaban represented the only DOAC. This uniformity minimises confounding from differing pharmacokinetic properties among anticoagulant agents.

Although DOACs are often prescribed in fixed doses, clinical guidance recognises the need to consider patient weight and renal function in certain populations, particularly in those at the extremes of bodyweight.^3,4^

In our cohort, one patient receiving edoxaban required a dose reduction after their postoperative weight fell below 60kg, demonstrating that postoperative weight change can influence even ‘fixed-dose’ regimens and emphasising the importance of early postoperative review of anticoagulant therapy.

Patients undergoing AKA lost substantially more weight (mean 8.7kg) than those undergoing BKA (mean 3.8kg), reinforcing the need for routine postoperative weight monitoring and timely medication review, particularly following higher-level amputations.

The limited number of anticoagulant dose adjustments observed in this study likely reflects a gap in routine postoperative assessment rather than an absence of clinical need. In many cases, patient weight is not remeasured during the inpatient recovery period, particularly when patients are medically stable or transferred early to rehabilitation or community facilities. Some patients remain bed-bound postoperatively due to clinical deterioration or intubation, making weight assessment impractical. Others await mobilisation before an accurate weight can be obtained – a process that may take several days or even weeks. During this interval, anticoagulant dosing often continues to be based on preoperative weight, potentially resulting in under- or overdosing under the false assumption that the patient’s bodyweight remains unchanged.

At present, there is no clear guidance in standard protocols – such as the BNF – that specifically advises repeat dose calculations following limb loss and the associated change in bodyweight.^7^

Postoperative care after major amputation should therefore be multidisciplinary, incorporating surgical, medical, physiotherapy and nutritional expertise. Routine monitoring of bodyweight and medication reconciliation should form part of this integrated care model to ensure patient safety and treatment efficacy. Weight monitoring is relevant not only for anticoagulant therapy but also for other weight-dependent medications, including insulin, antibiotics and certain analgesics.

We recommend that the weight of the amputated limb be measured intraoperatively and subtracted from the preoperative weight to estimate an early postoperative weight. This estimation can guide initial medication dosing until an accurate weight measurement is formally obtained.

This study has several limitations. It was retrospective, involved a small sample size and was conducted at a single vascular centre, which may limit the generalisability of the findings. Reliance on clinical records for weight documentation introduces potential under-reporting and measurement variability. Another key limitation is the absence of postoperative clinical outcomes data such as bleeding or thrombotic events, which precludes assessment of the direct clinical consequences of dosing discrepancies. Despite these limitations, the results underscore the need for greater awareness and further investigation into the pharmacological implications of weight change following amputation.

Future prospective work should aim to establish evidence-based guidelines for postoperative medication dose reassessment after major amputation and to define clinically relevant thresholds of weight change that warrant formal review.

## Conclusion

Major lower limb amputation is associated with significant physiological changes, including postoperative weight loss, which can affect the dosing accuracy of weight-dependent medications such as LMWH and DOACs. Although only a small proportion of patients in this cohort required dose adjustments, the potential consequences of inappropriate anticoagulant dosing – whether subtherapeutic or supratherapeutic – are clinically important. These findings emphasise the need for routine postoperative weight monitoring and medication review, particularly in high-risk vascular patients. Incorporating intraoperative measurement of amputated limb weight and using this to estimate immediate postoperative weight may support timely dose reassessment until a formal weight can be obtained. Further prospective studies are warranted to validate these findings and to inform evidence-based guidelines for postoperative anticoagulant management.

## Competing interests

The author/s declare no competing interests.

## Funding

The author/s received no financial support for the research, authorship and/or publication of this article.

## Ethics approval and consent to participate

Not applicable.

## Author contributions

**A Al-Kassar:** Conceptualisation, Data curation, Formal analysis, Investigation, Methodology, Project administration, Supervision, Validation, Writing – original draft, Writing – review & editing. **A Okram:** Data curation, Formal analysis, Investigation, Methodology, Writing – review & editing. **K Poluru:** Data curation, Investigation, Methodology, Writing – review & editing. **NB Teo:** Conceptualisation, Investigation, Methodology, Project administration, Supervision, Writing – review & editing.

## Artificial Intelligence

The authors declare that AI was used to only to improve the clarity, grammar and structure of the revised manuscript and to help format the response to reviewers. No part of the study design, data collection, statistical analysis or interpretation of results was conducted using AI tools. All scientific content and conclusions are entirely the authors’ own.

## Authorship notes

All authors were involved and contributed to the contributions to the conception, and design of the work, including interpretation of data for the work writing the draft and revision.

The abstract was previously orally presented in Paris Vascular Insight Conference, December 2023, Paris, France.
